# The Microbial Composition of Lower Genital Tract May Affect the Outcome of *in vitro* Fertilization-Embryo Transfer

**DOI:** 10.3389/fmicb.2021.729744

**Published:** 2021-10-01

**Authors:** Ruiying Wang, Guojun Zhou, Lukanxuan Wu, Xin Huang, Yujing Li, Bin Luo, Huili Zhu, Wei Huang

**Affiliations:** ^1^Department of Obstetrics and Gynecology, West China Second University Hospital of Sichuan University, Chengdu, China; ^2^Key Laboratory of Birth Defects and Related Diseases of Women and Children, Ministry of Education, Chengdu, China

**Keywords:** microbiota, vagina, cervix, *in vitro* fertilization, embryo transfer, infertility, pregnancy

## Abstract

**Objective**: This work was conducted in order to study the effect of the lower genital tract (vaginal and cervical canal) microbiota on pregnancy outcomes of reproductive-aged women receiving embryo transfer.

**Study design**: A total of 150 reproductive-aged patients who received the first fresh *in vitro* fertilization-embryo transfer (IVF-ET) were included in the study. Samples from the vagina and cervical site of each patient were collected separately using sterile swabs before ET. Genomic DNA was pyrosequenced for the V3–V4 regions of the 16S ribosomal RNA gene. Further bioinformatics analysis was performed using QIIME and R package. Pregnancy outcomes were followed and analyzed to compare differences in microbial composition.

**Results:** The cervical microbiota had a higher Shannon index than the vaginal microbiota, and the microbial composition was different between the two sites. However, the Sorenson index between the two sites within the same individual was 0.370 (0.309–0.400). A total of 89 patients achieved clinical pregnancy after ET, while 61 failed. The Shannon indices and the microbial community of both vaginal and cervical microbiota between pregnant and non-pregnant groups were not significantly different. The relative abundance of *Lactobacillus* in the vagina and cervical canal did not differ between the two groups. Linear discriminant analysis, random forest analysis, and receiver-operating characteristic curve analysis showed that *Bifidobacterium*, *Prevotella*, and *Lactobacillus iners* in the vagina, as well as *Solanum torvum*, *Fusobacterium*, and *Streptococcus* in the cervix, may be negatively associated with clinical pregnancy after IVF.

**Conclusion:** The cervical microbiota was more diverse than the vaginal microbiota, but because of anatomical continuity, there was a correlation between the two sites. The microbial composition of the vagina and cervical canal may influence the outcome of IVF-ET, but more samples are needed to verify this conclusion.

## Introduction

Infertility is a global public health and social issue and has affected about 48.5 to 72.4 million couples worldwide (Boivin et al., 2007; Mascarenhas et al., 2012). The main causes of infertility include tubal and pelvic factors, anovulation, male factors, and unexplained infertility. *In vitro* fertilization-embryo transfer (IVF-ET) is currently used as an effective assistive reproductive therapy for infertility (Stewart et al., 2011). However, many patients fail to conceive even after IVF-ET, possibly due to endometrial factors and poor embryo quality, but some patients experience unexplained implantation failure.

Microorganisms inhabit all organs of the human body in huge orders of magnitude (Sender et al., 2016). The Human Microbiome Project revealed significant differences in diversity and abundance of the human microbiota even in healthy individuals (Human Microbiome Project Consortium, 2012). The urogenital tract represents 9% of the whole human microbiota (Peterson et al., 2009). Recent advancements in high-throughput sequencing technology and the increase in microbial studies have shed light on the microbiota of the reproductive tract. Microbial communities of the vagina, cervical canal, uterus, fallopian tubes, and peritoneal fluid in women of reproductive age have been identified. This revealed the bacterial colonization of the upper genital tract and a continuum of microbiota along the reproductive tract (Chen et al., 2017). The presence of a microbial continuum emphasizes the importance of healthy microbiota in the reproductive process (Franasiak and Scott, 2015). Thus, the role of genital tract microbiota in embryo implantation has attracted immense attention in recent times.

A number of studies suggested that vaginal dysbiosis may have a negative impact on the outcome of IVF-ET. Collection of vaginal secretions prior to ET revealed that women with *Lactobacillus*-dominated vaginal microbiota were more likely to get pregnant (Bernabeu et al., 2019; Riganelli et al., 2020). Women with lower proportions of *Lactobacillus crispatus* in the vagina had a decreased chance of successful pregnancy, and the vaginal microbiota could be a good predictor of IVF outcomes (Haahr et al., 2019a; Koedooder et al., 2019). An imbalance in the vaginal microbiota, as in bacterial vaginosis, may reduce clinical pregnancy rates in IVF patients (Haahr et al., 2016). Moreover, vaginal microbial composition, especially the relative abundance of *Lactobacillus*, may be associated with recurrent implantation failure (RIF; Fu et al., 2020). However, some studies also found no effect of vaginal microbiota on pregnancy or live birth rates after IVF treatment (Haahr et al., 2019b; Vergaro et al., 2019). Whether and how vaginal microbiota affects the outcome of ET remains to be elucidated.

For cervical microbiota, earlier studies based on bacterial culture identification showed decreased implantation and clinical pregnancy rates in the group with a positive culture of *Escherichia coli* and *Streptococcus* on the tip of the transplanted catheter as well as the cervical canal swab (Egbase et al., 1996; Fanchin et al., 1998). Infertile women had fewer *Lactobacillus* and a higher diversity index in the cervical microbiota than fertile women (Graspeuntner et al., 2018). Several studies have also investigated the effects of the upper reproductive tract microbiota on ET. Some studies have suggested that intrauterine microbiota, such as *Lactobacillus*, might have an impact on embryo implantation and pregnancy maintenance (Moreno et al., 2016). Another study found that although *Lactobacillus* was the dominant genus, there was no link between *Lactobacillus* and pregnancy outcome in IVF patients (Franasiak et al., 2016). Riganelli et al. found that *Kocuria dechangensis* was significantly more abundant in non-pregnant women undergoing IVF (Riganelli et al., 2020). The role of the uterine microbial composition in reproductive outcomes remains controversial.

There are only limited high-throughput sequencing studies on the impact of cervical microbiota on ET. The correlation between cervical and vaginal microbiota of patients undergoing IVF-ET has also been less studied. Cervical mucus sampling can be used to investigate the uterine microenvironment prior to ET due to unfeasible endometrial sampling and the intraindividual correlation between microbiota in each reproductive tract site (Chen et al., 2017). Therefore, a study on the impact of vaginal and cervical microbiota on ET in a large sample of IVF patients is needed. In this study, we characterized the microbial composition in the vagina and cervical canal samples from infertile women who underwent IVF, and explored the potential genera contributing to pregnancy failure based on 16S rRNA gene amplicon sequencing.

## Materials and Methods

### Study Population

Infertile patients undergoing IVF or intracytoplasmic sperm injection (ICSI) due to tubal factor, endometriosis, ovulatory disorder, unexplained infertility, or male factors (i.e., severe oligoasthenoteratozoospermia and obstructive azoospermia) were recruited from the Department of Reproductive Medicine of West China Second University Hospital from June 2020 to November 2020. Patients aged 20–35years who received fresh cleavage-stage ET in the first IVF/ICSI cycle were enrolled in this study. Women with a history of assisted reproductive technology (ART); uterine malformations; mental diseases; hereditary diseases; uncontrolled endocrine disorders such as diabetes mellitus, hyperthyroidism, hypothyroidism, and hyperprolactinemia; severe systemic or organic diseases that affect maternal health; antibiotic or probiotic treatments within 2weeks before ET; current sexually transmitted or infectious diseases; and gynecological malignancy were excluded from the study.

A total of 150 infertile patients met the inclusion and exclusion criteria. Among them, 107 had tubal factors, 32 had ovulatory disorder, 16 had endometriosis, 3 had unexplained infertility, and 58 had male factors that led to infertility. They were treated with a gonadotropin-releasing hormone (GnRH) antagonist protocol, luteal phase short-acting long protocol, follicular phase long-acting long protocol, prolonged protocol, ministimulation protocol, or flare-up protocol for controlled ovarian stimulation.

Recombinant human chorionic gonadotropin (r-hCG) 10,000IU was used as a trigger to stimulate the final oocyte maturation when the diameter of at least two follicles ≥18mm. Oocyte retrieval was performed 36h after r-hCG administration. The day 3 cleavage-stage embryo grading was referred to the 2011 ESHRE Istanbul Consensus (Alpha Scientists in Reproductive Medicine and ESHRE Special Interest Group of Embryology, 2011), including cell number, fragmentation (%), symmetry, multinucleation, vacuoles, and zona pellucida. At least one of the embryos for transfer was a good-quality embryo of grade 7A, 8A, 9A, 7B, 8B, and 9B. ET was performed under the guidance of a transabdominal ultrasound. Each patient was followed up, and clinical pregnancy was confirmed by fetal cardiac activity on ultrasound at 35days after ET. Those who achieved clinical pregnancy were assigned to the pregnant group, while the others were assigned to the non-pregnant group.

This study was approved by the Ethics Committee of West China Second University Hospital of Sichuan University on May 21, 2019 (No. 2019–048), and all subjects signed informed consent forms before participating in the study.

### Sample Collection

Samples of the vagina and cervical canal were taken from each patient on the day of ET before the operation. The patient was placed in the lithotomy position. The vaginal sample was taken with a sterile swab from the upper third of the vagina. The surgeon wiped out secretions on the cervical os using sterile gauze, and then collected the cervical sample by rolling swabs gently inside the cervical canal. All sample swabs were placed inside 2ml Falcon tubes immediately, labeled, and stored in liquid nitrogen within 30min. They were then transferred to the laboratory and preserved at −80°C.

### Total DNA Extraction and 16S rRNA Sequencing

The total genomic DNA of each sample was extracted using a DNeasy PowerSoil kit (Cat. No. 12888, Qiagen, Hilden, Germany) according to the instructions of the manufacturer. The concentration of DNA was measured using NanoDrop2000. The V3–V4 variable regions of the 16S rRNA gene were amplified with universal primers 343\u00B0F (5'-TACGGRAGGCAGCAG-3'') and 798 R (5'-AGGGTATCTAATCCT-3'; Nossa et al., 2010) on a Bio-Rad 580BR10905 PCR instrument. PCR was performed with 15μl 2×Gflex PCR buffer, 1μl 5pmol/μl primer F, 1μl 5pmol/μl primer R, 50ng template DNA, 0.6μl Tks Gflex DNA Polymerase (1.25U/μl), H_2_O, and at a final reaction volume of 30μl. The PCR instrument was set up according to the following conditions: denaturation at 94°C for 5min, 26cycles of denaturation at 94°C for 30s, annealing at 56°C for 30s, and elongation at 72°C for 20s, and a final extension at 72°C for 5min. Amplicons were purified with Agencourt AMPure XP beads (A63881, Beckman, Brea, CA, United States). The final amplicons were quantified using the Qubit dsDNA Assay kit (Q32854, Thermo Fisher Scientific, Waltham, MA, United States). Equal amounts of purified amplicons were pooled for subsequent sequencing. Sequencing was performed using an Illumina NovaSeq 6000 platform.

### Microbiota Analysis

Paired-end reads were filtered using Trimmomatic software (version 0.35; Bolger et al., 2014). Ambiguous bases (N) and low-quality sequences were detected and cut off. After trimming, paired-end reads were assembled using FLASH software (version 1.2.11; Reyon et al., 2012). The sequences were then further denoised. Reads with ambiguous, homologous sequences or those below 200bp were abandoned. Reads with 75% of the bases above Q20 were retained. Then, reads with chimeras were detected and removed using UCHIME (Edgar et al., 2011). Further quality control and chimera checks were performed using the QIIME software (version 1.8.0; Caporaso et al., 2010). Valid tags were clustered into operational taxonomic units (OTUs) using Vsearch software (version 2.4.2) with a 97% similarity threshold (Rognes et al., 2016). The representative read of each OTU was selected using the QIIME package, and then assigned and annotated against the Greengenes database using the RDP classifier (Wang et al., 2007). The α-diversity represented by the Shannon–Wiener index was calculated in QIIME. The analysis of β-diversity was calculated based on the weighted UniFrac distance matrix algorithm and represented by principal coordinates analysis (PCoA). The difference in β-diversity between the two groups was calculated using the permutational multivariate analysis of variance (PERMANOVA) test of the R package vegan. The Sorenson index was calculated to evaluate similarity in the same individual. To count the species with significant differences between groups, Metastat analysis was used, and linear discriminant analysis (LDA) was performed using Galaxy software. The top 30 genera in terms of relative abundance were taken, and the R package randomForest was used to map the genera importance points.

### Statistical Analysis

Continuous variables were expressed as medians (25–75 interquartile range) or mean±SD, and categorical variables were expressed as numbers or relative frequencies (%). The Kolmogorov–Smirnov test was used to normalize the distribution. Student’s *t*-test or Mann–Whitney *U* test was performed to compare quantitative variables. Parametric or non-parametric tests were used for variable distribution. The χ^2^ test was performed to compare the categorical variables. The receiver-operating characteristic (ROC) curve was used to calculate the cutoff value and the area under the curve for the assessment of the predictive value of specific genera. All analyses were performed using the Statistical Package for Social Sciences (SPSS version 25.0, Chicago, IL, United States). A two-tailed value of *p*<0.05 was considered statistically significant.

## Results

### Characterization of Vaginal and Cervical Microbiota

A total of 150 infertile patients that matched the inclusion criteria were enrolled in the study. Based on their clinical pregnancy results, 89 were included in the pregnant group and 61 failed pregnancies were included in the non-pregnant group. The characteristics of the participants are shown in Table 1.

**Table 1:** Clinical characteristics of the participants and comparisons between groups according to the achievement of clinical pregnancy.

Clinical characteristics	Pregnant group (*N*=89)	Non-pregnant group (*N*=61)	*P*-value
Age (years)	30 (27–31)	30 (27.5–32)	0.773
BMI (kg/m^2^)	21.94±2.78	21.49±2.65	0.409
AMH (ng/ml)	2.37 (1.53–4.02)	2.84 (1.58–4.01)	0.560
Duration of infertility (years)	2 (1–4)	3 (1–5)	0.156
**Cause of infertility (n)**			
Tubal factor	59	44	0.449
Ovulatory disorder	18	14	0.689
Endometriosis	11	5	0.417
Unexplained	2	1	1.000
Male factor	31	27	0.244
**Gravity**			
No	57	32	0.156
Yes	32	29	
**Parity**			
No	83	54	0.311
Yes	6	7	
**Hormone stimulation**			
GnRH-antagonist protocol	40	33	0.639
Luteal phase short-acting long protocol	30	18	
Follicular phase long-acting long protocol	14	10	
Prolonged protocol	3	0	
Ministimulation protocol	1	0	
Flare-up protocol	1	0	
E_2_ on the HCG-day (pg/ml)	1,996.2 (1,302.8–2,777.7)	2,200.7 (1,721.2–2,806.2)	0.188
Retrieved oocytes (*n*)	8 (6–12)	8 (6–11)	0.319
**IVF or IVF-ICSI**			
IVF	67	46	0.986
IVF-ICSI	22	15	
Endometrial thickness (cm)	1.1 (1.0–1.2)	1.1 (0.95–1.2)	0.639
**ET**			
SET	8	6	0.861
DET	81	55	

Values are expressed as median (25–75 interquartile range), mean ± SD, and number.

AMH, anti-Müllerian hormone; IVF, in vitro fertilization; ICSI, intracytoplasmic sperm injection; ET, embryo transfer; SET, single embryo transfer; DET, double embryo transfer; GnRH, gonadotropin-releasing hormone; E_2_, estradiol.

**Figure 1:**
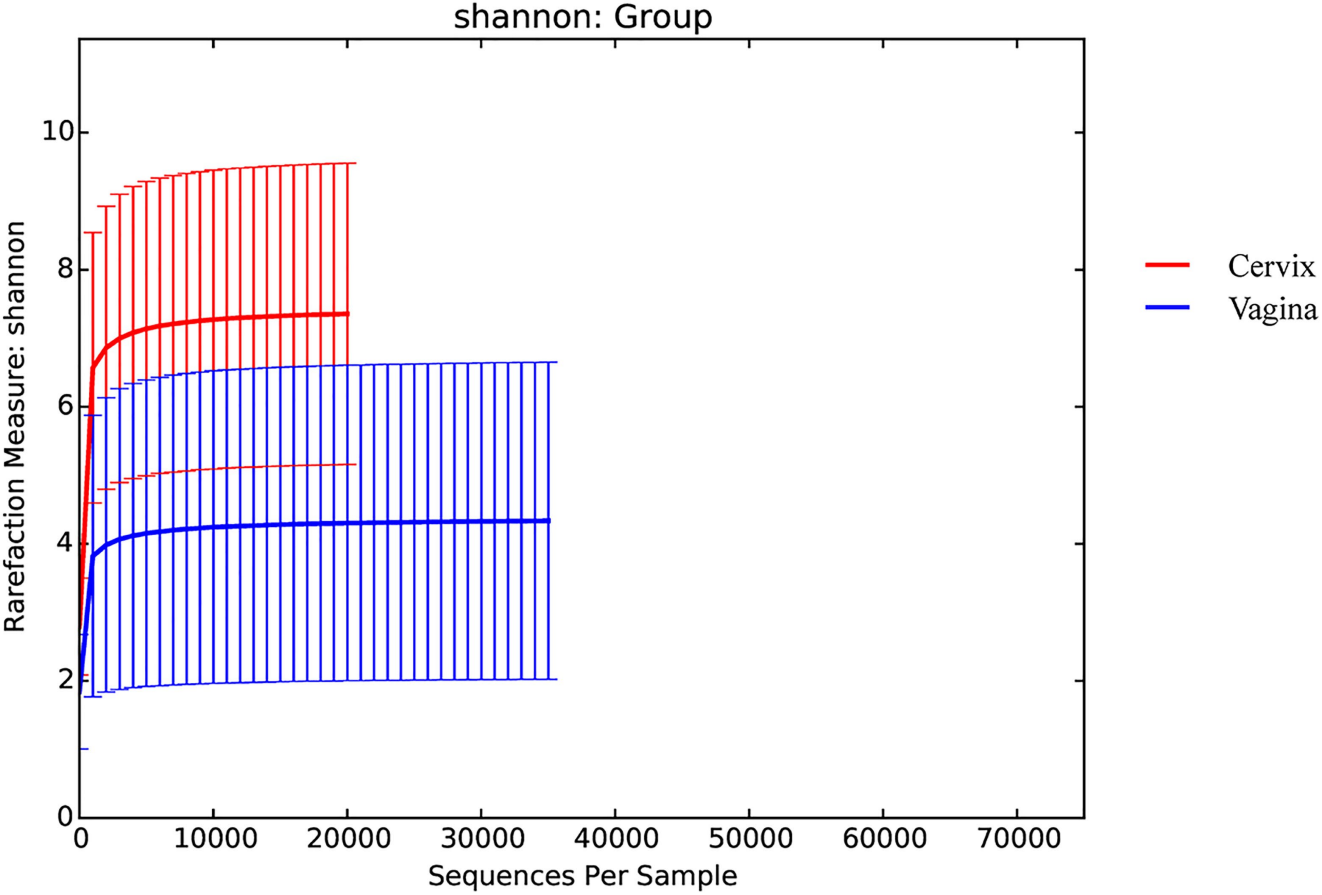
Rarefaction curves for the Shannon index for each group.

There were 9,600,715 total sequences for the 150 vaginal samples (mean 65,219; range 35,195 to 74,022). There were 8,542,141 total sequences for the 150 cervical samples (mean 59,604; range 20,667 to 72,494). The rarefaction curves for the Shannon diversity index of each group demonstrated that the sequencing depth was sufficient (Figure 1).

**Figure 2:**
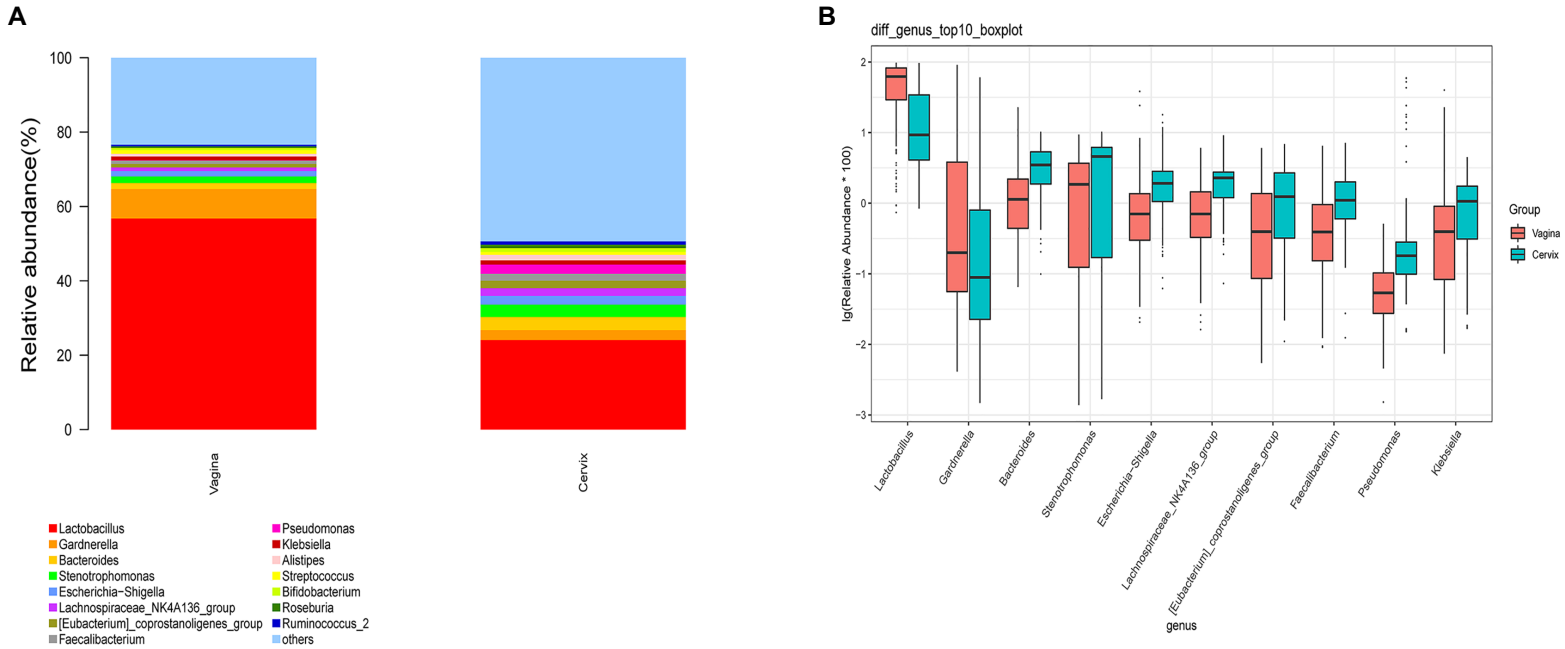
The microbial composition of cervix and vagina and the different genera between the two sites. **(A)** The relative abundance of the top 15 genera at the genus level in the vaginal and cervical microbiota. **(B)** Boxplot of the top 10 genera in the differential genera between two groups.

A total of 29,462 OTUs were identified in the vaginal microbiota. The microbial composition of the vaginal microbiota is displayed in Figure 2A (left column). *Lactobacillus* was the most abundant genus (56.80% of the microbial composition), followed by *Gardnerella* (7.93%), *Stenotrophomonas* (1.75%), *Bacteroides* (1.60%), *Escherichia-Shigella* (1.49%), *Klebsiella* (1.03%), *Streptococcus* (1.03%), *Lachnospiraceae_NK4A136_group* (1.0%), and others. For vaginal samples, 12% (18/150) were *Lactobacillus*-dominated (>90% *Lactobacillus*), while 42.67% (64/150) had more than 70% of the *Lactobacillus* genus.

A total of 35,755 OTUs were identified in the cervical microbiota. In the cervical microbiota (Figure 2A, right column), *Lactobacillus* was the most abundant genus (24.08% of the microbial composition), followed by *Bacteroides* (3.57%), *Stenotrophomonas* (3.32%), *Gardnerella* (2.66%), *Pseudomonas* (2.49%), *Escherichia-Shigella* (2.33%), *Lachnospiraceae_NK4A136_group* (2.10%), and others. For cervical samples, only 2.67% (4/150) were *Lactobacillus*-dominated (>90% *Lactobacillus*), while 9.33% (14/150) had more than 70% of the *Lactobacillus* genus.

**Figure 3:**
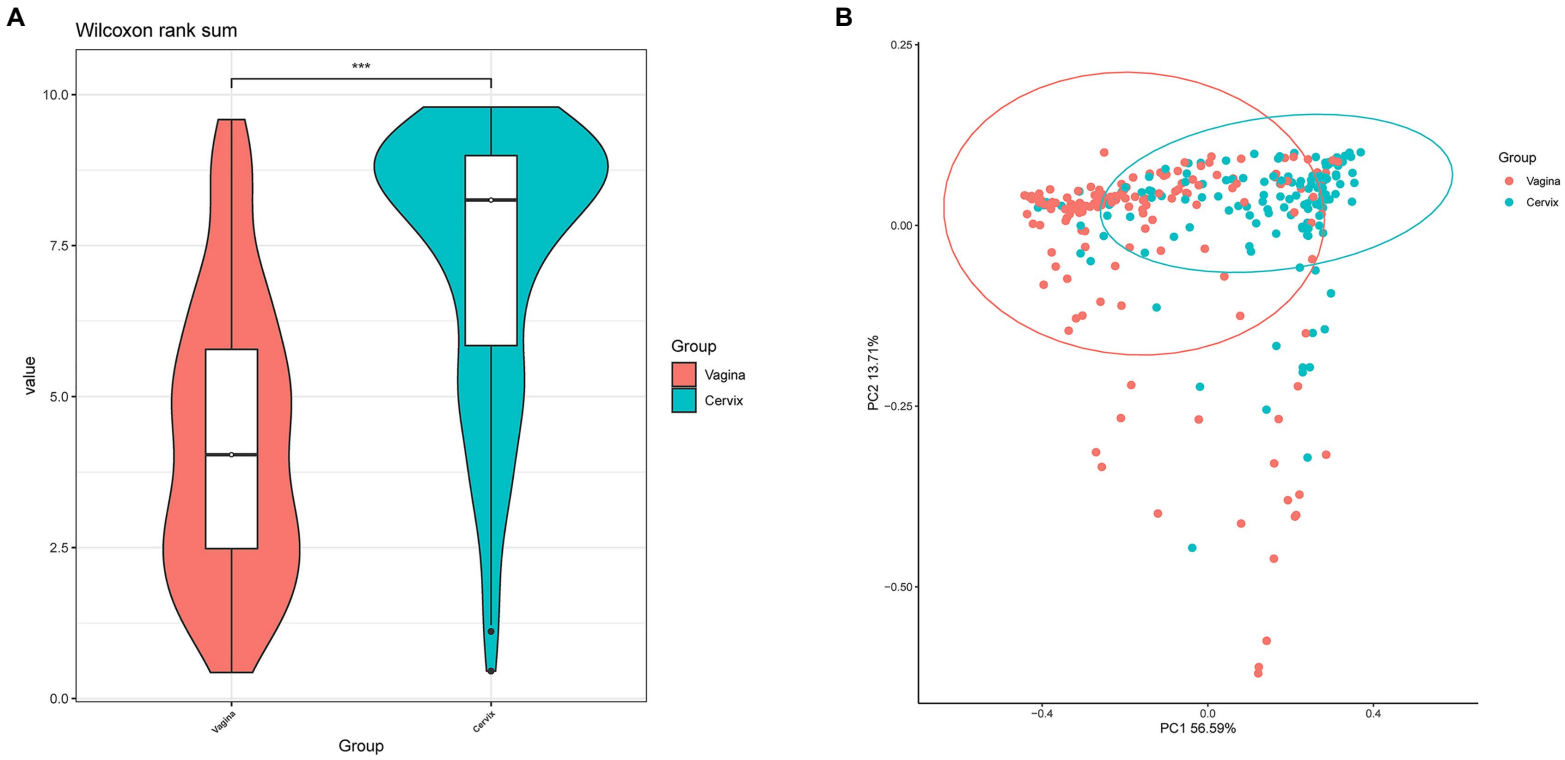
Differences between cervical and vaginal microbiota. **(A)** The Shannon diversity indices of vaginal and cervical microbiota. **(B)** A principal coordinates analysis (PCoA) based on weighted UniFrac distance metrics was applied to represent the distribution of the vaginal and cervical microbial communities.

**Figure 4:**
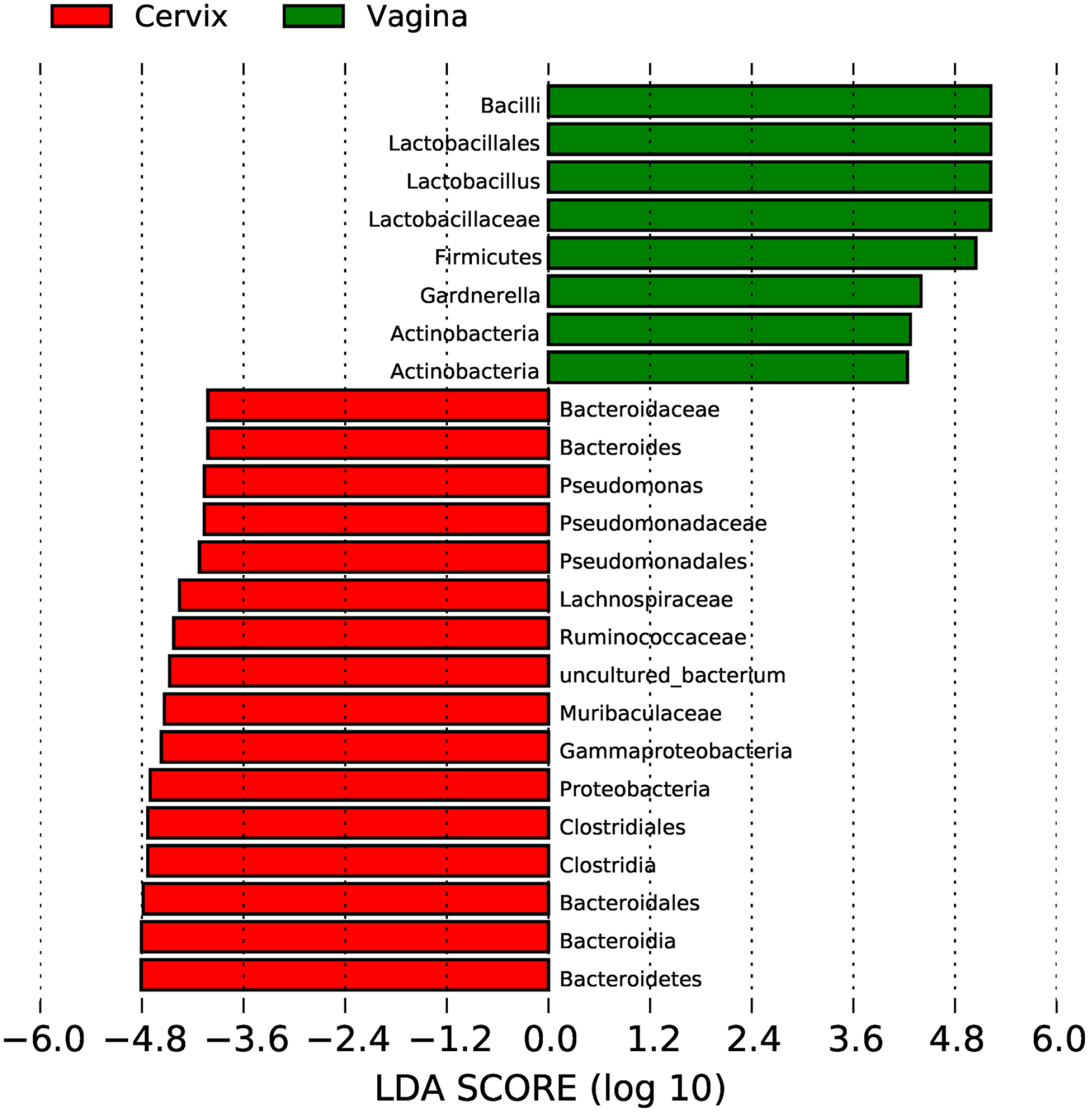
Linear discriminant analysis to explore potential genera contributing to intergroup differentiation. The green bar indicates the genera contributing to the vaginal microbiota, while the red bars indicate the genera contributing to the cervical microbiota.

*Lactobacillus* and *Gardnerella* were the primary genera in both the vagina and cervical canal, but they both comprised a greater proportion in the vagina (Figure 2B, *p*<0.001). However, other abundant genera were more abundant in the cervix (Figure 2B). The Shannon diversity index of the cervical microbiota (8.25, 5.69–9.00) was higher than that of the vaginal microbiota (4.04, 2.47–5.81, *p*<0.01; Figure 3A). PCoA based on weighted UniFrac distance metrics between the cervix and vagina revealed significantly different clustering (Figure 3B, *p*<0.001). LDA also suggested that *Lactobacillus* and *Gardnerella* were significant in the vagina, while *Pseudomonas* and *Bacteroides* contributed to the cervical microbiota (Figure 4). To examine the intraindividual relationship of the cervical and vaginal microbiota, we computed the intraindividual Sorenson index of 0.370 (0.309–0.400) for the same individual. Samples from the same individuals were also correlated.

### Comparison of the Vaginal and Cervical Microbiota Between Pregnant and Non-pregnant Groups

**Figure 5:**
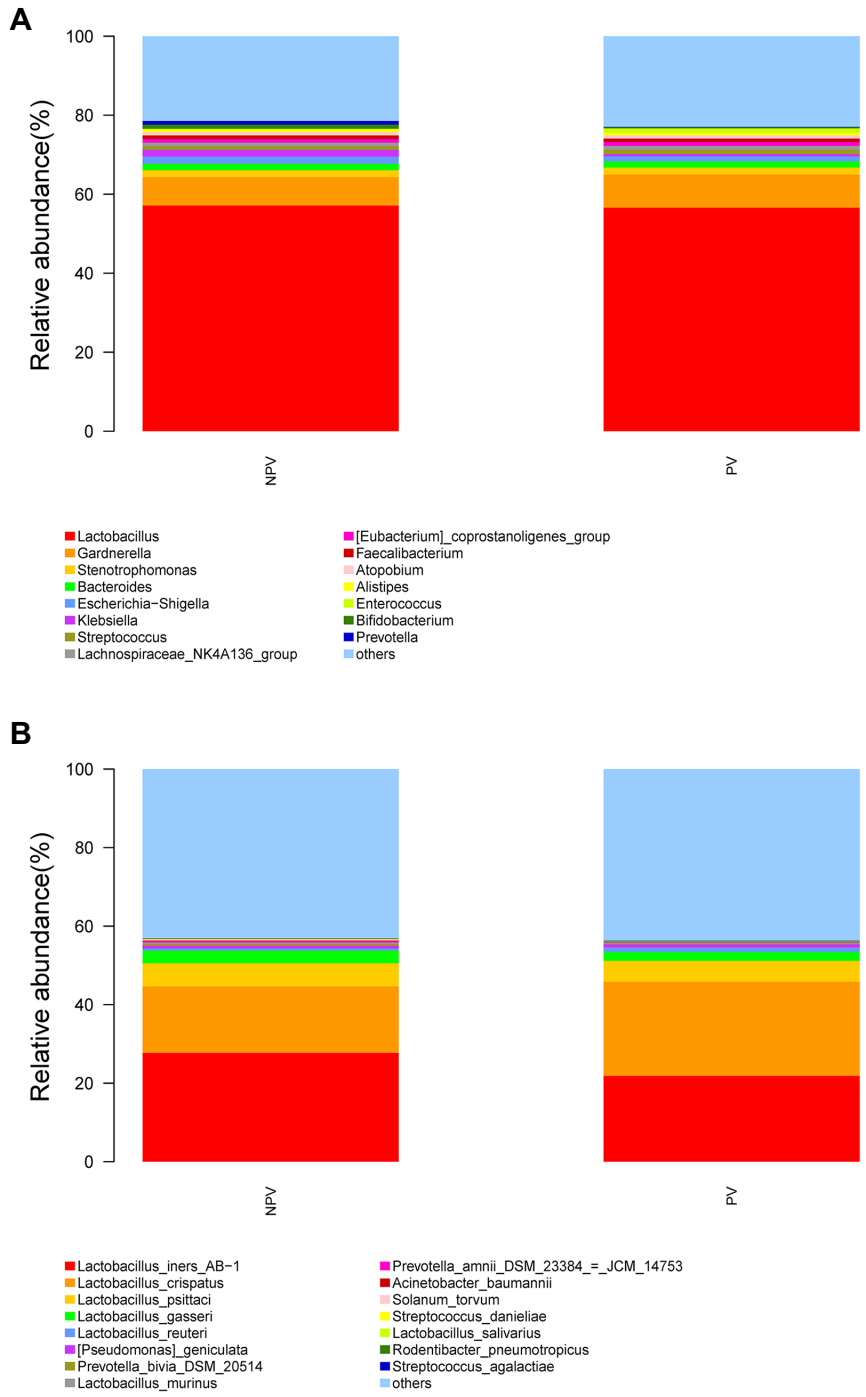
Vaginal microbial composition of pregnant and non-pregnant groups. The relative abundance of the top 15 genera in the vagina at the genus level **(A)** and species level **(B)** in pregnant and non-pregnant groups.

**Figure 6:**
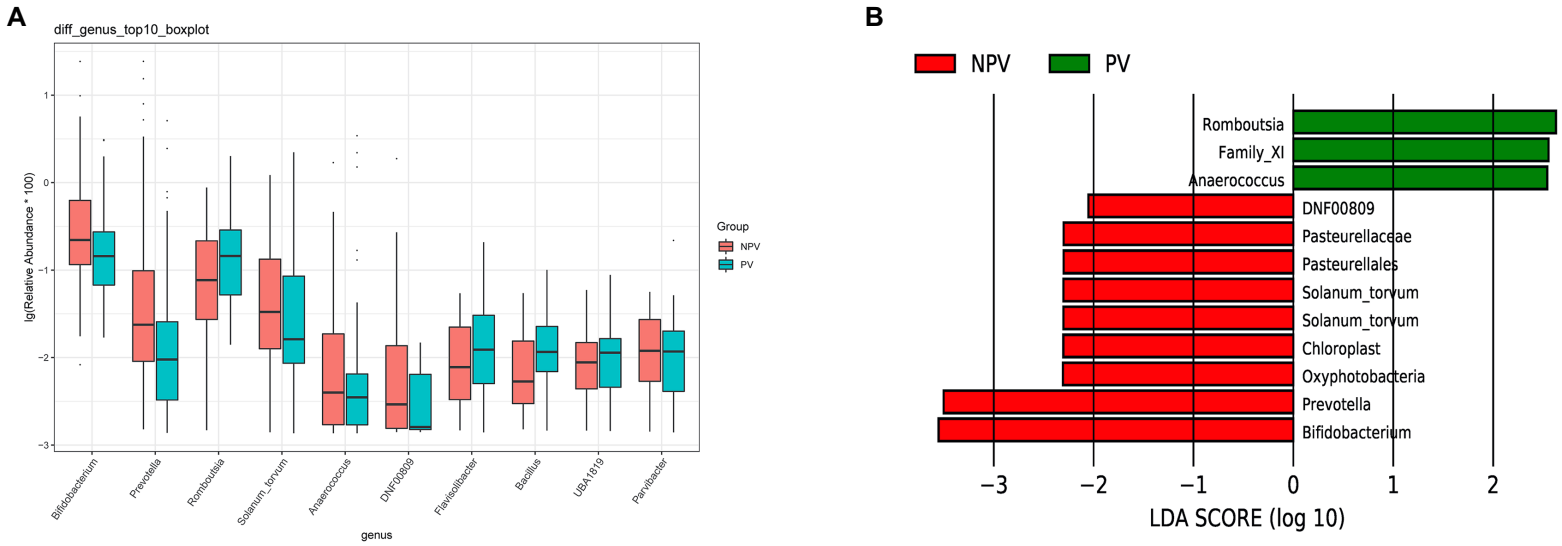
Differences in the vaginal microbiota between pregnant and non-pregnant groups. **(A)** Boxplot of the top 10 genera in the differential genera between the two groups. **(B)** Linear discriminant analysis to explore potential genera in vagina contributing to intergroup differentiation. The green bar indicates the genera contributing to the pregnant group, while the red bars indicate the genera contributing to the non-pregnant group.

**Figure 7:**
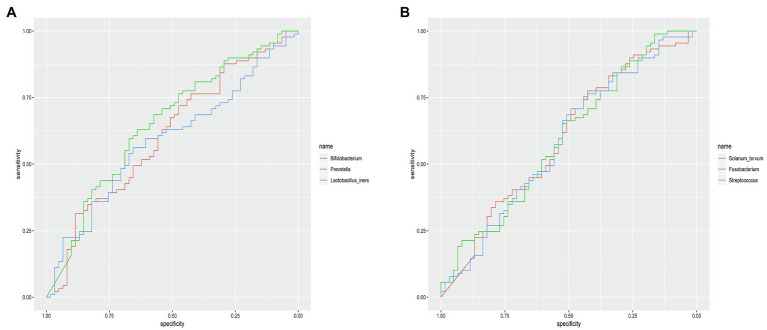
ROC curves of specific genera for predicting non-pregnancy after IVF treatment. **(A)** ROC curves of *Prevotella*, *Bifidobacterium*, and *Lactobacillus iners* in the vagina for predicting non-pregnancy after IVF. **(B)** ROC curves of *Solanum torvum*, *Fusobacterium*, and *Streptococcus* in the cervix for predicting non-pregnancy after IVF.

The microbial composition of the vagina at the genus level appeared to be similar between pregnant and non-pregnant groups (Figure 5A). The Shannon indices (*p*=0.67) and microbial community structure of the vagina (*p*=0.838) were comparable between the two groups. At the species level, *L. crispatus* and *Lactobacillus iners* were the most abundant *Lactobacillus* species (Figure 5B). The relative abundance of *L. iners* in the pregnant group (21.90%) was lower than that in the non-pregnant group (27.75%, *p*=0.069), while *L. crispatus* was more abundant in the pregnant group (23.97%) than that in the non-pregnant group (16.91%, *p*=0.183), but the differences were not statistically significant. Additionally, there were 55 genera in the vaginal microbiota that were significantly different between the two groups, and the top 10 genera are shown in Figure 6A. The proportion of *Bifidobacterium* was 1.00% in the non-pregnant group and 0.29% in the pregnant group (*p*=0.026). *Prevotella* was more abundant in the non-pregnant group (0.99%) than that in the pregnant group (0.12%, *p*=0.004). Meanwhile, *Bifidobacterium* and *Prevotella* were the special genera contributing to non-pregnancy based on LDA (LDA score>3; Figure 6B). ROC curve analysis showed that *Prevotella* (AUC=0.651, *p*=0.002) and *Bifidobacterium* (AUC=0.609, *p*=0.024) were predictive of a non-pregnancy outcome after IVF. For *L. iners*, the AUC was 0.589 (*p*=0.064) and the cutoff value was 5.69% (Figure 7A). The pregnancy rate of patients with a relative abundance of *L. iners*≥5.69% was 49.37% (39/79), lower (*p*=0.009) than that in patients with a relative abundance of *L. iners*<5.69% (50/71, 70.42%).

**Figure 8:**
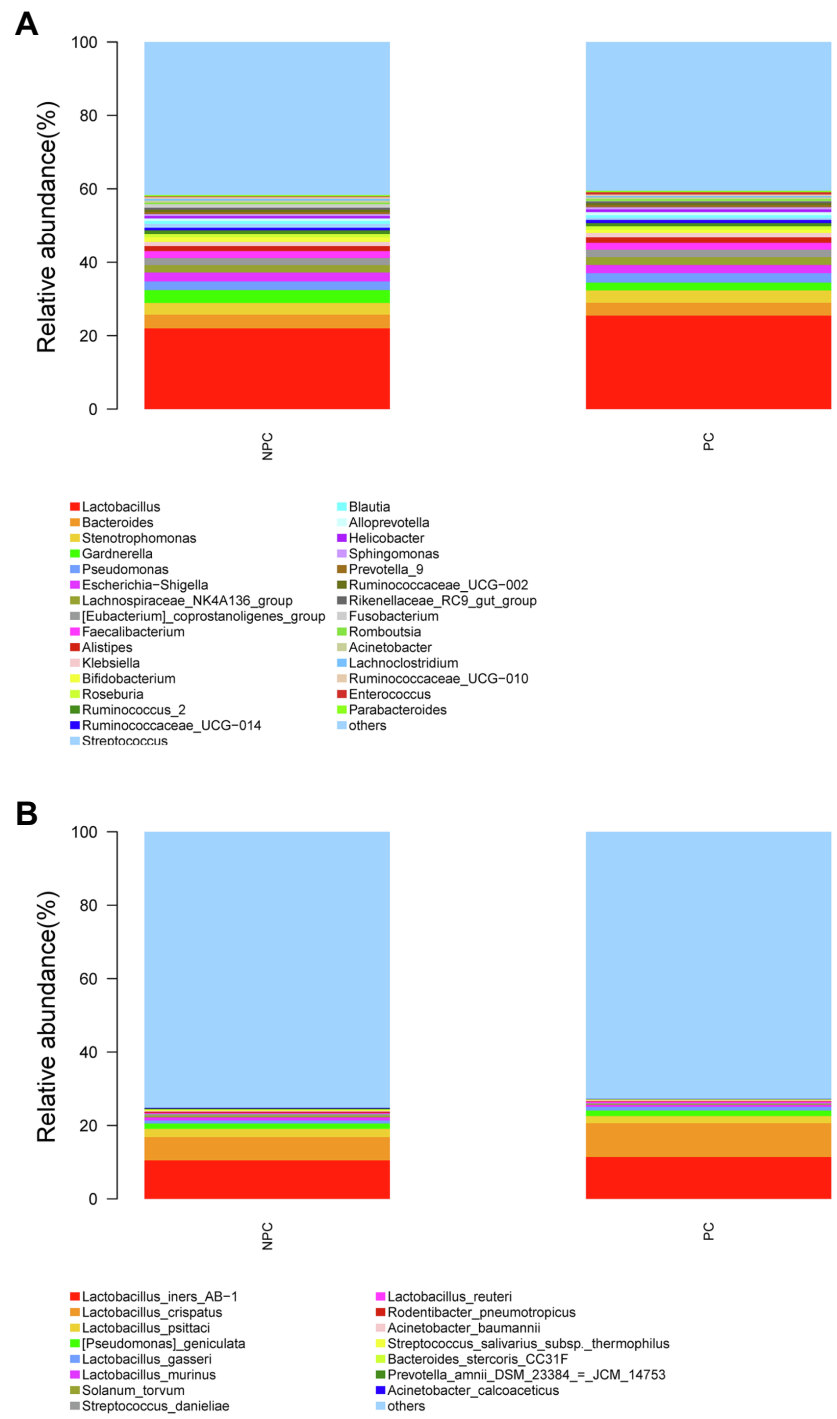
Cervical microbial composition of pregnant and non-pregnant groups. **(A)** The relative abundance of the top 30 genera in the cervical canal at the genus level in pregnant and non-pregnant groups. **(B)** The relative abundance of the top 15 genera in the cervical canal at the species level in pregnant and non-pregnant groups.

**Figure 9:**
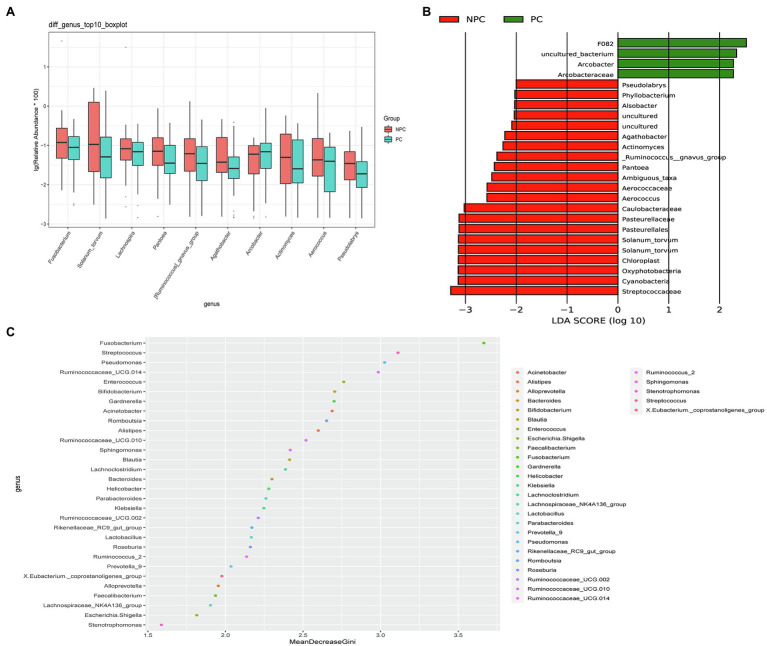
Differences in the cervical microbiota between pregnant and non-pregnant groups. **(A)** Boxplot of the top 10 genera in the sdifferential genera between the two groups. **(B)** Linear discriminant analysis to find potential genera contributing to intergroup differentiation. The green bar indicates the genera contributing to the pregnant group, while the red bars indicate the genera contributing to the non-pregnant group. **(C)** Random forest analysis for mapping genera importance points. The horizontal coordinate is the importance measure, and the vertical coordinate is the name of the genera after ranking by importance.

The microbial composition of the cervix in the pregnant and non-pregnant groups revealed similar patterns (Figure 8A). *Lactobacillus*, *Bacteroides*, *Stenotrophomonas*, *Gardnerella*, and *Pseudomonas* did not differ between the two groups. At the species level, *L. crispatus* was more abundant in the pregnant group (9.23%) than that in the non-pregnant group (6.30%), although the difference was not statistically significant (*p*=0.074, Figure 8B). There were 74 genera that were significantly different between the two groups, and the top 10 genera are shown in Figure 9A. *Solanum torvum* and *Fusobacterium* had higher relative abundances in the non-pregnant group. *S. torvum* contributed mostly to a negative pregnancy outcome based on LDA (LDA score>3; Figure 9B). According to random forest analysis, *Fusobacterium* and *Streptococcus* were found to be the potential genera for predicting the occurrence of a negative pregnancy outcome after ET (Figure 9C). The pregnancy rate of patients with a relative abundance of *Streptococcus*≥1% was 44.74% (17/38) and decreased (*p*=0.034) in patients with a relative abundance of *Streptococcus*<1% (72/112, 64.29%). ROC curves showed that *S. torvum* (AUC=0.596, *p*=0.047) and *Fusobacterium* (AUC=0.596, *p*=0.047) were predictive of a negative pregnancy outcome (Figure 7B).

## Discussion

This study included 150 women who underwent their first fresh cycle of IVF or ICSI, and compared the vaginal and cervical microbial composition between pregnant and non-pregnant patients to explore the effect of lower genital microbial composition on the outcome of ET.

In our study, we found that many genera were abundant in both the cervix and vagina, and the Sorenson index confirmed intraindividual correlation between cervical and vaginal microbiota in the same patient. The similarity of cervical and vaginal microbiota in infertile patients undergoing IVF was related to the anatomical location of the cervix and vagina, and the colonizing microbes might migrate between the two sites. However, a higher Shannon index suggested a more diverse cervical microbiota. The PCoA plot also demonstrated that the microbial communities in the cervix and vagina were significantly different. The vaginal microbiota was dominated by *Lactobacillus*, resulting in a significant decrease in diversity. The variation in the microbial composition between the two sites may be due to the blocking of cervical mucus leading to changes during migration and colonization, or changes in the environment caused by large amounts of lactic acid produced by abundant *Lactobacillus* in the vagina (Aldunate et al., 2015).

Ravel et al. clustered vaginal microbial communities into five groups, known as community state types (CSTs). CST I, II, III, and V were dominated by *Lactobacillus*, while CST IV included more abundant strictly anaerobic bacteria (Ravel et al., 2011). *Lactobacillus*, one of the dominant genera in the vaginal microbiota, is capable of producing lactic acid (Aldunate et al., 2015) and bacteriocins (Barbés and Boris, 1999) to maintain a healthy environment and prevent pathogen invasion. The relative abundance of *Lactobacillus* has been suggested to be related to infertility (Zhao et al., 2020) and RIF (Fu et al., 2020). In our study, *Lactobacillus* and *Gardnerella*, although abundant, might have no effect on the outcome of ET. This result is similar to that of a previous study (Bernabeu et al., 2019). *Lactobacillus crispatus* and *L. iners* are the main *Lactobacillus* species in the vagina (Ravel et al., 2011). A previous study suggested that *L. crispatus* might have a positive impact on pregnancy rates (Koedooder et al., 2019; Vergaro et al., 2019), and *L. iners* was more likely associated with vaginal dysbiosis (Petrova et al., 2017). Similar to previous studies, we observed that *L. crispatus* was more abundant and *L. iners* was less abundant in the pregnant group. Although the differences were not statistically significant, we found a trend. *L. iners*≥5.69% was negatively correlated with pregnancy. *L. iners*, as the abundant species in the vagina, may be indicative of non-pregnancy. Larger samples may be needed to verify the positive effects of *L. crispatus* on pregnancy.

Some studies suggested that vaginal microbiota did not affect live birth rates or clinical pregnancy rates (Haahr et al., 2019b; Vergaro et al., 2019). A previous study found that *Bifidobacterium* might have a negative effect on pregnancy (Zhao et al., 2020). *Prevotella* is anaerobic and it has been shown to be associated with increased levels of cytokines, such as IL-1β, IL-8, and IL-17 (Larsen, 2017). In our study, we found an increase in *Bifidobacterium* and *Prevotella* in the vaginal microbiota of patients who failed to conceive, and they were also indicative of non-pregnancy. Vaginal microbial composition, such as the increase in *Prevotella*, *Bifidobacterium*, and *L. iners*, might play an important role in embryo implantation.

Fanchin et al. (1998) reported that positive cultures of *E. coli* and *Streptococcus* in the cervical microbiota on ET catheters led to decreased pregnancy rates. Salim et al. (2002) found a significant association between cervical colonization and pregnancy achievement. In our study, we found that an increase in *S. torvum*, *Streptococcus*, and *Fusobacterium* might be associated with conception failure. Meanwhile, it is possible that *L. crispatus* in the cervical microbiota could be favorable for the achievement of pregnancy, but the correlation was not confirmed. The inconsistency between our study and previous studies on the effect of cervical and vaginal microbiota might be due to the study population, sample size, research method, or sequencing fragments. However, *S. torvum*, *Streptococcus*, and *Fusobacterium* in the cervix, as well as *Bifidobacterium* and *Prevotella* in the vagina, were not so abundant. In particular, the ROC curve suggested that the predictive value of *S. torvum* and *Fusobacterium* in the cervix for non-pregnancy is not very high. Meanwhile, the mechanism of their influence on pregnancy outcomes after IVF was not determined in this study. More appropriate samples are therefore needed to validate their negative association with pregnancy and whether they affect pregnancy outcomes through metabolites (Fu et al., 2020) or immune factors (Benner et al., 2018).

Previous studies have confirmed that ovulation induction had no effect on the vaginal microbiota of infertile women who underwent IVF (Zhao et al., 2020). In this study, we fixed the sampling on the day of day 3 cleavage-stage ET in the fresh cycle to minimize the effect of ART protocols on the genital microbiota for convincing results. Although baseline parameters such as age, duration of infertility, ART protocols, embryo quality, and endometrial thickness did not differ between pregnant and non-pregnant groups in this study, there were large within-group differences due to differences in microbiota between individuals. A larger multicenter study is needed to validate these results. In addition, a larger sample will allow subgroup analysis of patients with biochemical pregnancy or abortion to investigate the effects of reproductive microbiota on early embryo implantation failure and spontaneous abortion after clinical pregnancy.

With the development of sequencing technology, the impact of reproductive tract microbial profiles on human reproduction has been increasingly well understood. This study explored the effect of the lower genital tract microbiota on fresh ET. *Prevotella*, *Bifidobacterium*, and *L. iners* in the vagina, as well as *S. torvum*, *Streptococcus*, and *Fusobacterium* in the cervical canal, may affect embryo implantation and have predictive value for IVF-ET outcomes. However, studies on the microbiota in the upper genital tract are lacking. Therefore, more large-scale studies on the microbiota of the reproductive tract continuum and its impact on ET, as well as interventional treatments such as probiotic or antibiotic therapies to improve the microecology of the reproductive tract, are needed.

## Data Availability Statement

The datasets presented in this study can be found in online repositories. The names of the repository/repositories and accession number(s) can be found at: https://www.ncbi.nlm.nih.gov/bioproject/PRJNA741994.

## Ethics Statement

The studies involving human participants were reviewed and approved by the Ethics Committee of West China Second University Hospital of Sichuan University. The patients/participants provided their written informed consent to participate in this study.

## Author Contributions

RW and GZ designed the study and collected the samples. RW analyzed the data and wrote the manuscript. LW, XH, and YL contributed to the collection, arrangement, and transfer of the samples. BL provided statistical advice and contributed to the revision of the manuscript. HZ contributed to the collection of samples and guided the study. WH designed and guided the study and revised the manuscript. All authors read and approved the final version of the manuscript for submission.

## Funding

This study was supported by a grant from West China Second University Hospital of Sichuan University (KL029).

## Conflict of Interest

The authors declare that the research was conducted in the absence of any commercial or financial relationships that could be construed as a potential conflict of interest.

## Publisher’s Note

All claims expressed in this article are solely those of the authors and do not necessarily represent those of their affiliated organizations, or those of the publisher, the editors and the reviewers. Any product that may be evaluated in this article, or claim that may be made by its manufacturer, is not guaranteed or endorsed by the publisher.
